# Physiological and behavioral reactions elicited by simulated and real-life visual and acoustic helicopter stimuli in dairy goats

**DOI:** 10.1186/1746-6148-7-16

**Published:** 2011-04-15

**Authors:** Franz Josef van der Staay, Martin Joosse, Henk van Dijk, Teun Schuurman, Jan van der Meulen

**Affiliations:** 1 BioMedical Research, Wageningen University and Research Center, Lelystad, The Netherlands; 2 National Aerospace Laboratory, Amsterdam, The Netherlands; 3 Program Emotion & Cognition, Department of Farm Animal Health, Veterinary Faculty, University Utrecht, Utrecht, The Netherlands; 4 Rudolf Magnus Institute of Neuroscience, University Utrecht, Utrecht, The Netherlands

## Abstract

**Background:**

Anecdotal reports and a few scientific publications suggest that flyovers of helicopters at low altitude may elicit fear- or anxiety-related behavioral reactions in grazing feral and farm animals. We investigated the behavioral and physiological stress reactions of five individually housed dairy goats to different acoustic and visual stimuli from helicopters and to combinations of these stimuli under controlled environmental (indoor) conditions. The visual stimuli were helicopter animations projected on a large screen in front of the enclosures of the goats. Acoustic and visual stimuli of a tractor were also presented. On the final day of the study the goats were exposed to two flyovers (altitude 50 m and 75 m) of a Chinook helicopter while grazing in a pasture. Salivary cortisol, behavior, and heart rate of the goats were registered before, during and after stimulus presentations.

**Results:**

The goats reacted alert to the visual and/or acoustic stimuli that were presented in their room. They raised their heads and turned their ears forward in the direction of the stimuli. There was no statistically reliable rise of the average velocity of moving of the goats in their enclosure and no increase of the duration of moving during presentation of the stimuli. Also there was no increase in heart rate or salivary cortisol concentration during the indoor test sessions. Surprisingly, no physiological and behavioral stress responses were observed during the flyover of a Chinook at 50 m, which produced a peak noise of 110 dB.

**Conclusions:**

We conclude that the behavior and physiology of goats are unaffected by brief episodes of intense, adverse visual and acoustic stimulation such as the sight and noise of overflying helicopters. The absence of a physiological stress response and of elevated emotional reactivity of goats subjected to helicopter stimuli is discussed in relation to the design and testing schedule of this study.

## Background

It has been suggested that flyover of aircrafts and helicopters at low altitude may elicit a stress- and anxiety-related physiological and behavioral reaction in grazing farm animals. Similarly, the noise produced by traffic, tractors, or machinery at short distance and high intensity may impair animal welfare [1].

### Noise

Noise is an unwanted sound, either chronic or intermittent from a variety of sources in the environment [2]. Aircrafts produce significant amounts of sound, the majority of which is produced from turbojet engines, but helicopters are also a source of severe low frequency sound and vibration [3]. Animal species vary greatly in their response to noise, depending on the animal's hearing ability, duration of the noise, type of habitat, time of day and year, the activity the animal is engaged in at the time of exposure, sex and age, level of any previous exposure and whether other physical stresses are present [4]. Despite these variable factors, most researchers agree that noise does have an effect on animal physiology and behavior. These effects can potentially lead to problems in animal's general health and long-term survival [2,4].

### Goat hearing and sight

Goats have a well-developed hearing and sight [5]. The hearing of goats ranges from 78 Hz to 37 kHz with a well-defined point of best sensitivity at 2 kHz. The sound localization acuity of goats is relatively poor [6]. Thresholds for brief complex sounds in a two-choice procedure averaged 18° and 30° around the median sagittal plane for cattle and goats, while in the same test apparatus the threshold for humans and dogs averaged 0.8° and 8° respectively. Like other poor localizers, cattle and goats are prey species with their best vision directed throughout nearly the entire horizon [6]. In contrast to mammals with very narrow foveal fields, they do not need very accurate locus information from their auditory systems to direct their gaze to a sound source [6].

### Fear and anxiety

Anxiety is characterized as a response to potential danger [7], as generalized distress independent from a specific stimulus [8]. Fear has been characterized as a response to present danger [7], in other words, as stimulus or cue-specific [8,9]. Fear primes the organism to act reflexively in response to these stimuli or cues. The primary locus of control are sub-cortical structures such as the amygdala that activates hypothalamic nuclei that activate the sympathetic autonomic nervous system and the hypothalamic-pituitary-adrenal (HPA) axis. In parallel, higher brain regions such as the hippocampus and the cerebral cortex may be activated ("bottom-up", [7]; see also [8,9]). However, almost all activated neuronal capacity is focused toward the immediate threat, severely interfering with normal processing of information [7].

**Figure 1:**
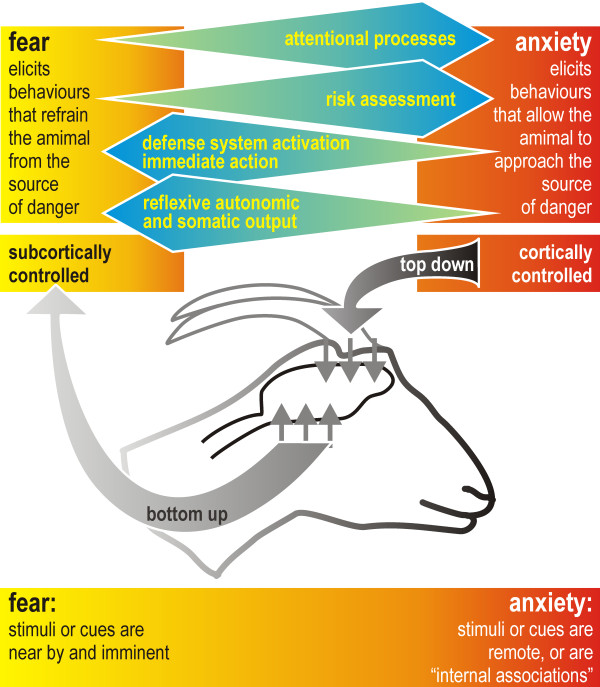
**A conceptual distinction between fear and anxiety**. Fear is controlled sub-cortically, whereas higher brain regions control anxiety [modified from [13]].

Anxiety elicits behaviors that enable the animal to approach the source of (perceived) threat [10] by increasing attention and stimulating risk assessment [8,11]. The frontal cortex is the primary locus of control; it processes the perceived threat cognitively, and is able to modulate and steer lower levels of neuronal processing ("top-down", [7]; see also [8,9]). In particular, risk assessment may be distorted in anxiety. Anxiety-driven hyper-activity of the hippocampus, which has a central role in cognitive processing and inhibitory functions, may have a central role in disturbed cognition [12,13]. Figure 1 depicts the distinction between fear and anxiety schematically, based on the proximity and specificity of the threatening stimuli, and on the primary locus of control (sub-cortical versus cortical). In general fear responses are less easy to modulate than anxiety responses [14,15].

### Reactions of feral goats to aircraft and helicopter noise

A review of the sparse published information on ruminants suggests that their reactions show characteristics of strong fear which is controlled subcortically, and which activates the HPA axis. The observation that these physiological and behavioral reactions can be strengthened by negative experiences with helicopters (e.g. shooting from the helicopter), providing evidence for the notion that learning (which is predominantly a cortical process) may modulate this reaction (Figure 1) [13].

In Australia helicopters are used to measure the abundance of free-ranging feral goats (*Capra hircus*) [5]. The typical response in 90% of the feral goats to a helicopter flying overhead was an increased alertness, usually immediate fright and alert bolting, followed by alert moving and standing. Finally, they returned to pre-disturbance, non-alert, activities. The percentage of alert goats decreased exponentially with helicopter distance, with an average of 20% still alert when the helicopter was 2.5 km away. The incidence and extent of alert activity and the distance moved in response to a helicopter varied between herds, maybe related to habituation to anthropogenic disturbances [5]; feral goats that had experience with shooting from a helicopter showed a much earlier reaction [16], most likely as a consequence of learning. The structure of groups of goats following helicopter flyover was disturbed in 42% of all occasions.

The goats showed a learned alert response to the helicopter over time by moving more often but over shorter distances with subsequent flyovers, possibly into areas that they perceived as 'safe'. In response to flyovers and despite a minimum of 3-month time lag between helicopter samplings, goats retreated to, and congregated at the same locations within their home range. This was possibly due to social facilitation, where the detection of a threat by a few alert animals showing a greater alert response resulted in the whole group moving towards a nearby refuge. The aversive response of feral goats is likely complex; a learned, socially facilitated, rapid alert response with movement behavior directed towards a familiar refuge [5].

The noise and the optical appearance of a helicopter are usually undifferentiated in studies on helicopter disturbance of wildlife. The likely stimulus causing animals to flush is noise, as there was no significant effect of direct sighting of the helicopter on mountain goat (*Oreamnos americanus*) reactions once the effect of distance of mountain goats to the helicopter was accounted [17]. The decrease in alert behavior with distance from the helicopter and alert responses of feral goats when helicopters were invisible to them also indicates that auditory cues are dominant, but do not rule out the impact of visual cues [5]. However, purely visual stimuli may also cause panic flights, as has been reported for the Alpine ibex (*Capra ibex*) in response to hang gliding or paragliding in the Alpines [18].

### Aims of the study

Goats, although domesticated approximately 10,000 years ago, are still poorly studied farm animals [19]. The reactions of feral goats to the sound and sight of a helicopter may be poor predictors for the behavior of dairy goats. They are usually well habituated to human approach and to the sight and noise of traffic (both airborne and terrestrial) and other machinery. Systematic investigations of the reaction of dairy goats to the presentation of strong stimuli such as helicopters flying over are still missing. The present study was conceived to close this gap.

We investigated the behavioral and physiological stress reactions of five individually housed dairy goats to different acoustic and visual stimuli from a tractor passing by, or a helicopter flying over under controlled environmental (indoor) conditions. The stimuli were tractor or helicopter animations projected on a large screen in front of the enclosures of the goats. Acoustic and visual stimuli were presented either apart or in combination. On the final day of the study the goats were exposed to two flyovers (altitude 50 m and 75 m) of a Chinook helicopter while grazing in a pasture. Salivary cortisol, behavior, and heart rate of the goats were registered before, during and after stimulus presentations.

## Methods

### Ethical note

The study was reviewed and approved by the local ethics committee (DEC, **d**ier**e**xperimenten **c**ommissie) under number 2008136.a, and was conducted in accordance with the recommendations of the EU directive 86/609/EEC. All effort was taken to minimize the number of animals used and their suffering.

### Animals

Five non-pregnant, non-lactating white Dutch dairy goats with an average age of 50.8 (±0.7) weeks (range 49 - 53 weeks) and a height of withers of approximately 70 cm were purchased from a commercial dairy goat farm. The goat farm lies in an area in which helicopters are allowed to fly at low altitude. All animals were healthy and in excellent physical condition.

### Housing

The goats were transported to the Animal Sciences Group in Lelystad, The Netherlands. They were housed in a room measuring 18.25 m × 12.00 m that contained 6 enclosures of large-meshed gauze measuring 1.85 m × 2.50 m with an interspace of approximately 1.00 m. The enclosures were lined in two rows of 3 enclosures. The middle enclosure in the first row remained empty. The goats were housed individually in the other five enclosures. The temperature (average 18°C) and humidity (average 43%) controlled room was illuminated by white fluorescent strip lights (lights on from 6:00 to 22:00). The goats were fed twice daily with food pellets (standard goat food pellets) and hay. Water was always available *ad libitum*.

A video camera (Sony TC506C) was mounted above each enclosure. A large projection screen (2 m × 3 m) was positioned in front of the two rows of enclosures. Visual stimuli could be projected onto this screen by a beamer (Dell 3300MP) that was fixed to the ceiling above the empty enclosure. Hidden behind the projection screen stood a large speaker box (Raveland CM150). A microphone (PCB type 377B02 type 1/n 101276) fixed to the ceiling above the second row of enclosures at a height of 2.5 m registered the sound emitted by the speaker. The signal was preprocessed by a data acquisition card (National Instruments MI4472) and fed into a personal computer. Volume and frequency of the acoustic stimuli, as registered by the microphone, were continuously compared with the intended setpoint and readjusted if necessary. The computers and all additional equipment needed to calibrate and control the stimulus presentations and to register the behavioral and physiological responses of the goats were hidden behind a shielding made of black tarpaulin in the same room that housed the goats.

### Procedures

All animals were allowed to habituate to housing in separate enclosures, the presence of the animal caretakers outside and inside the enclosures, a thoracic girdle, and to the procedure for collecting saliva using a cotton bud.

#### Stimulus presentations

The behavioral and physiological reactions of the animals to different acoustic and visual stimuli and their combinations were assessed on four days of testing (Table 1) following the two-week habituation period. Stimuli were presented for 3 minutes. Observation of the goat's behavior and heart rate were started 12 minutes before the presentation of the stimuli and terminated 12 minutes after cessation of the stimuli. A cycle of seven stimulus presentations was run on days 1 and 2. On day 3, 8 stimulus presentations were given. The projected tractor and helicopter were animated, except the 8^th ^stimulus presentation on day 3 (no. 22; Table 1), where the projection of a video recording of an Apache helicopter that approached and flew away served as stimulus.

**Table 1:** Stimuli presented and time points of saliva sampling

	Stimulus	Day	Time	Visual	Acoustic	Salivary cortisol
			7:30			●
			8:50			●
	1	1	9:15	gray air		
			9:30			●
	2	1	10:00	helicopter 10%		
			10:15			○
	3	1	11:00	tractor 10%		
			11:15			○
**Visual stimuli**	4	1	11:45	helicopter 45%		
			12:00			○
			12:50			○
	5	1	13:15	tractor 45%		
			13:30			○
	6	1	14:00	helicopter 80%		
			14:15			○
	7	1	14:45	gray air		
			15:00			○
			15:15			○

			7:30			●
			8:50			●
	8	2	9:15			
			9:30			●
	9	2	10:00		helicopter sounds (85 dB; 30 - 1400 Hz)	
			10:15			○
	10	2	11:00		tractor sounds (85 dB; 50 - 1200 Hz))	
			11:15			○
**Acoustic stimuli**	11	2	11:45		helicopter sounds (90 dB; 30- 1400 Hz)	
			12:00			○
			12:50			○
	12	2	13:15		tractor sounds (90 dB; 50 - 1200 Hz)	
			13:30			○
	13	2	14:00		helicopter sounds (95 dB; 30 - 1400 Hz)	
			14:15			○
	14	2	14:45			
			15:00			○
			15:15			○

			7:30			●
			8:50			●
	15	3	9:15	gray air		
			9:30			●
	16	3	10:00	helicopter 10%	helicopter sound (85 dB; 30 - 1400 Hz)	
			10:15			○
	17	3	11:00	tractor 10%	tractor sound (85 dB; 50 - 1200 Hz)	
			11:15			○
**Visual and acoustic stimuli combined**	18	3	11:45	helicopter 45%	helicopter sound (90 dB; 30 - 1400 Hz)	
			12:00			○
			12:50			○
	19	3	13:15	tractor 45%	tractor sound (90 dB; 50 - 1200 Hz)	
			13:30			○
	20	3	14:00	helicopter 80%	helicopter sound (95 dB; 30 - 1400 Hz)	
			14:15			○
	21	3	14:45	gray air		
			15:00			○
			15:15			○
	22	3	15:35	video Apache helicopter, 40%	peak level 90 dB; 30 - 1400 Hz	
			15:50			○
			16:05			○

			12:00			●
	23	6	13:30			●
**Flyover Chinook helicopter**	24	6	13:45	Flyover Chinook at 75 m	peak level 100 dB; 40 - 600 Hz	
			13:55			○
	25	6	14:15	Flyover Chinook at 50 m	peak level 110 dB; 40 - 600 Hz	
			14:30			○
			15:00			○

If not indicated otherwise, the visual and acoustic stimuli were those provided by the environment in which the goats were kept (the experimental room on days 1 to 3 of testing, or grassland before, during and after flyover of the Chinook helicopter on day 6 of testing). The normal background noise ranged indoors from 58 dB to 70 dB, and outdoors from 68 dB to 75 dB. For the visual stimuli the percentages indicate the breadth of the tractor or helicopter projection with respect to the breadth of the projection screen. The numbers 1 to 25 (first column) are used in the figures of the results section to indicate which stimulus or combination of stimuli was presented. ●: Baseline cortisol measurements (averaged per day for statistical analysis); ○: Cortisol measurements following the first stimulus presentation of a day (averaged per day for statistical analysis).

The helicopter animation consisted of projecting a series of helicopter movements with the main and tail rotor of the helicopter rotating, each lasting 20 sec, separated by 20-second periods of projecting gray air. The movements were presented in this order: helicopter flew left to right at constant height; helicopter appeared in the top left corner, flew to the center of the screen, and disappeared in the upper right corner; helicopter approached frontally (increasing size) and flew back (decreasing size); helicopter appeared at the center of the upper edge of the screen, decreased to the center of the screen, and climbed again. In the tractor animation, the tractor moved slowly from the left to the right at the bottom edge across the projection screen. Key characteristics of the acoustic stimuli (maximum sound pressure level in decibels, and frequency range) that were presented alone or in combination with the visual stimuli are summarized in Table 1).

On the fifth day, the goats were transferred to a pasture where they were kept in enclosures of the same dimension as used in the experimental room. They were allowed to habituate to this new environment for about 4 hours before they were returned to the experimental room. No measurements were performed during the habituation session, and the goats were left undisturbed.

On the sixth day, the goats were again transferred to the pasture. They were provided the opportunity to habituate for approximately 2 hours. During the habituation period, equipment needed to register the goat's behavior was arranged around the pasture. Then, the goats were exposed to a Chinook helicopter, flying over twice at low altitude. The physiological and behavioral reactions of the goats were measured during and after both flyovers of the Chinook helicopter.

The normal stable environment, the projection of gray air during days 1 to 3, and the normal environment during pasture on the sixth day were considered as neutral stimuli.

### Heart rate

Heart rate was registered telemetrically using the Polar S810 system (Polar Elektro Oy, Finland). A thoracic girdle with two integrated electrodes, one positioned ventrally and one left laterally, and a transmitter for wireless data transfer was fixed behind the front legs. Electrode gel (Spectra 360, Parker) was used to improve conductivity. The receiver with data logger was fixed at the girdle on the back of the goat. After presentation of one or two stimuli, the data logger was detached and the data were downloaded onto a computer via an interface (Polar Precision Performance SW 4.00.023). Heart rates were calculated as beats per minute (bpm).

### Salivary cortisol

Saliva was collected at different time points preceding, during and after testing (Table 1). Saliva samples (approximately 1 mL) were collected by allowing the goats to chew on a cotton bud (Paul Hartmann, Nijmegen, the Netherlands) until the bud was moistened. The bud was then placed in special centrifuge tubes with inner cases (Sarstedt BV, Etten Leur, The Netherlands) and centrifuged for 10 min at 3000 rpm. The collected saliva was stored at -15°C. Salivary cortisol was measured by radioimmunoassay (Coat-a-Count Cortisol TKCO, Siemens Medical Solutions Diagnostics), performed according to the manufacturer's instructions. The detection limit was 0.16 ng.mL^-1 ^and the intra-assay coefficient of variation was 8.7%.

### Behavior

#### Behavior indoors

Before, during and after stimulus presentations on days 1-3, the behavior of the goats was registered using the automatic video tracking system EthoVision (Version 3.1.16, Noldus Information Technology, Wageningen, The Netherlands) [20,21] and stored in a digital video-recorder and on a computer as MPEG2 files, using MPEG recorder 2.0 (Noldus). The following measures were calculated: velocity of moving (cm.s^-1^), and percentage time moving during each of the observation periods before, during and after stimulus presentation.

#### Behavior outdoors

On the sixth day, the following behaviors of the goats were scored from the video recordings: lying, standing, walking, urination/defecation, running (attempts to escape), eating/drinking, and raised head with ears turned forward as index of alertness. The duration of locomotion was the sum of the time spent walking, running and attempting to escape. This measure was expressed as percent time moving of the respective observation periods.

#### Statistical analyses

For each stimulus presentation (no. 1 to 22 in Table 1) and for each of the 12 minutes preceding a stimulus presentation, the 3 minutes of stimulus presentation, and the 12 minutes after cessation of a stimulus presentation, the average heart rate, average velocity and average duration moving (expressed as percent of total per observation period) was determined.

Due to equipment failure, a few heart rate measurements were lost, and one average value could not be calculated for goat no. 5 on day 1 and 3 averages on the second day for goat no. 1. Consequently, the number of goats in the repeated measures ANOVA of these days was reduced to 4 for days 1 and 2. Note that for the means and standard errors of the means (SEM) all available data were used (i.e. most means represent the average heart rates of 5 goats).

On the sixth day, 2 averages of the heart rate measurements could not be calculated for goat no. 3 and only the alertness of three of the five goats could be scored reliably.

#### Baseline measurements

##### Cortisol

The means of the first two samples per day, i.e. the measurements preceding the first non-neutral stimulus presentations, were used to estimate baseline values. The stability of the baseline measurements was assessed by a repeated measures ANOVA with the factor Days.

##### Heart rate, velocity and percent time moving

The stability of the baselines of these three measures was determined by repeated measures ANOVAs on the values of the 12-minute periods preceding stimulus presentations, with the factor Stimulus test periods.

##### Effects of stimulus presentations

##### Cortisol

The means of all measures of a day that followed after the first two baseline measures served as indication for cortisol response to stimulus presentations. The effects of the stimulus presentations on salivary cortisol levels were analyzed per day by an ANOVA with the repeated measures factor Stimulus presentations (Mean of baseline cortisol vs. mean of cortisol response to stimulus presentation).

##### Heart rate, velocity and percent time moving

The effects of stimulus presentation were analyzed *per day *by an ANOVA with the repeated measures factors Stimulus test periods (the average of the 12-minute period before, 3-minute period during, and 12-minute period after stimulus presentation) and Observation periods (measurements in the periods before, during, and after stimulus presentation).

In addition, the difference scores between the averages of the observation periods 1) during - before stimulus presentation, 2) after - during stimulus presentation, and 3) after - before stimulus presentation were calculated. One-sample *t*-statistics were performed to analyze whether these difference scores differed from zero.

## Results

### Cortisol

**Figure 2:**
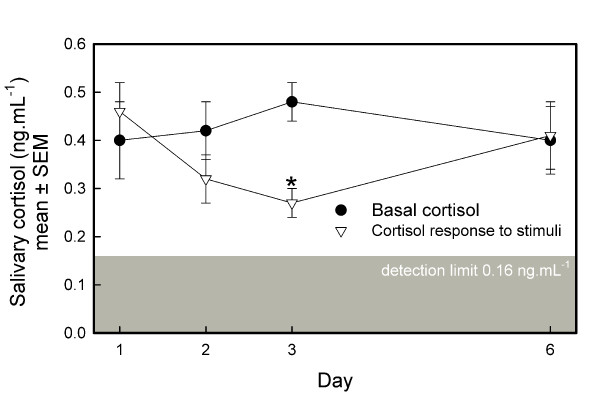
**Daily basal salivary cortisol level and salivary cortisol response to stimulus presentations of 5 dairy goats**. The daily baseline cortisol (ng.mL^-1^; mean ± SEM) was calculated as mean of the cortisol measurements preceding presentation of non-neutral stimuli (Table 1, last column), whereas the salivary cortisol response represents the mean of all cortisol measurements of a day, following the first stimulus presentation. *: One sample t-statistics on difference score between basal cortisol and cortisone response to stimuli, p < 0.05.

The baseline measurement of salivary cortisol revealed low values that did not change over the four days of testing (F_3,12 _= 0.35, p < 0.7919; Figure 2).

Comparing the cortisol levels at baseline with the average of all cortisol levels measured after the first stimulus was presented revealed that the average cortisol levels did not change over days (Days: F_3,12 _= 0.31, p < 0.8192), but tended to be affected by the stimuli (Stimulus presentation: F_1,4 _= 2.98, p < 0.1596; Days by Stimulus presentation interaction: F_3,12 _= 3.10, p < 0.0672). This was most probably due to a drop in cortisol levels after stimulus presentations on day 3 (one-sample t-statistics on difference score between the mean basal cortisol level minus the mean cortisol level of all measurements after the first stimulus presentation: t_4 _= 6.65, p < 0.0027).

### Heart rate

**Figure 3:**
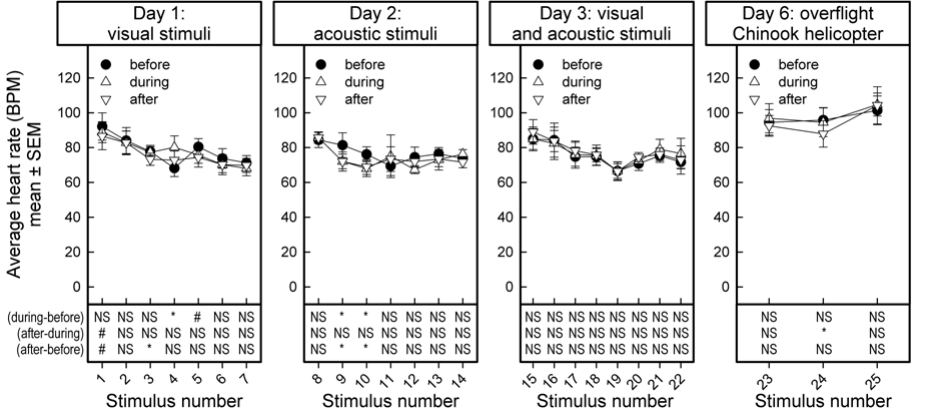
**Average heart rate before, during, and after stimulus presentation in 5 dairy goats**. Heart rate (beats per minute, BPM) is depicted as mean ± SEM. Note that during the overflight of the Chinook helicopter, only the heart-rates of four goats were registered reliably. The list of stimuli presented is shown in Table 1. Results of one-sample t-statistics on the difference scores between values (during minus before), (after minus during), and (after minus before) stimulus presentation are summarized in the lower part of the figures. NS: no effect, p > 0.10; #: marginal effect, 0.10 > p > 0.05; *: effect, p < 0.05

Stability of baseline measurements: The baseline measurements changed across stimulus presentations (F_24,72 _= 2.88, p < 0.0003; Figure 3), probably because the heart rate was, on average, slightly higher when the goats were tested on day 6 than of the other days of testing. The slight increase in heart rate may have been a response to transferring the animals to the pasture.

On the first day, the average heart rate was not different between the visual stimuli (Stimulus test periods: F_6,18 _= 2.05, p < 0.1096). The stimuli differentially affected the cardiac response (Observation periods: F_2,6 _= 0.86, p < 0.4684; Stimulus test periods by Observation periods interaction: F_12,36 _= 3.89, p < 0.0008). This effect was likely due to an increase in heart rate in response to presentation of stimulus 4, whereas no change in heart rate was observed in response to the presentation of the other stimuli.

Heart rates decreased slightly across the successive presentations of acoustic stimuli on day 2 (Stimulus test periods: F_6,18 _= 3.17, p < 0.0267). The stimuli affected the cardiac response (Observation periods: F_2,6 _= 3.35, p < 0.1506, Stimulus test periods by Observation periods interaction: F_12,36 _= 3.30, p < 0.0027) to presentation of stimuli 9 and 10 but not to other stimuli.

The heart rates slightly changed across the successive presentations of the combination of visual and acoustic stimuli (Stimulus test periods: F_7,28 _= 2.79, p < 0.0248). We did not observe changes in heart rate in response to the stimulus presentations (Observation periods: F_2,8 _= 0.86, p < 0.4579, Stimulus test periods by Observation periods interaction: F_14,56 _= 0.45, p < 0.9507).

### Observations

Although the behavior of the goats in the stable was not scored systematically on days 1 to 3 of the experiment, it was obvious that the goats were alert when the visual and/or acoustic stimuli were presented. They raised their heads and turned their ears forward in the direction of the stimuli.

### Average velocity moving

**Figure 4:**
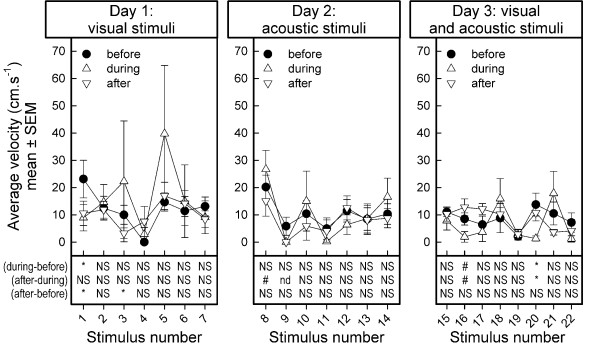
**Average velocity before, during, and after stimulus presentation in 5 dairy goats**. Velocity is depicted as mean ± SEM. Results of one-sample t-statistics on the difference scores between values during minus before, after minus during, and after minus before stimulus presentation are summarized in the lower part of the figures. The list of stimuli presented is shown in Table 1. NS: no effect, p > 0.10; nd: not determined, because all observations are zero-values, i.e. no goat was moving during a observation period; #: marginal effect, 0.10 > p > 0.05; *: effect, p < 0.05

Stability of baseline measurements: The baseline measurements changed across stimulus presentations (F_21,84 _= 2.48, p < 0.0019; Figure 4)).

Presentation of visual stimuli on day 1 had no effects on the velocity moving (Stimulus test periods: F_6,24 _= 0.99, p < 0.4520; Observation periods: F_2,8 _= 1.51, p < 0.2767; Stimulus test periods by Observation periods interaction: F_12,48 _= 0.73, p < 0.7193).

On the second day of testing, the velocity moving, averaged over the three observation periods of the acoustic stimulus presentations, changed across stimuli (Stimulus test periods: F_6,24 _= 3.97, p < 0.0067). However, the velocity moving was not affected by the stimulus presentations (Observation periods: F_2,8 _= 0.83, p < 0.4707; Stimulus test periods by Observation periods interaction: F_12,48 _= 1.09, p < 0.3904).

Velocities moving tended to differ between stimuli on the third day of testing (Stimulus test periods: F_7,28 _= 2.14, p < 0.0715), and the different combinations of visual and acoustic stimuli affected the velocity moving differently (Observation periods, F_2,8 _= 6.19, p < 0.0238; Stimulus test periods by Observation periods interaction: F_14,56 _= 2.15, p < 0.0222).

### Percent duration moving

**Figure 5:**
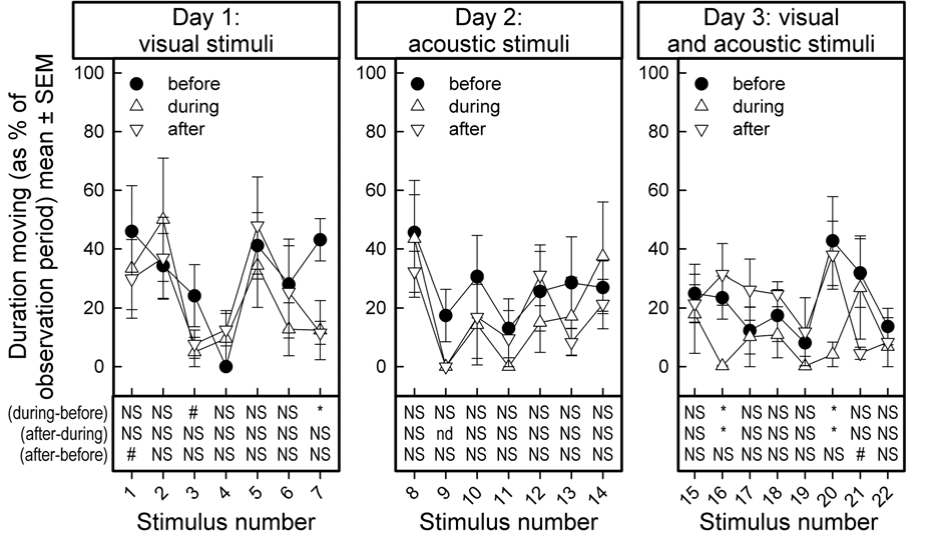
**Average duration moving, before, during and after stimulus presentation in dairy goats**. Duration moving is expressed as percent of the total observation period (mean ± SEM). Results of one-sample t-statistics on the difference scores between values (during - before), (after - during) and (after - before) stimulus presentation are summarized in the lower part of the figures. The list of stimuli presented is shown in Table 1. NS: no effect, p > 0.10; nd: not determined, because all observations are zero-values, i.e. no goat was moving during an observation period; #: marginal effect, 0.10 > p > 0.05; *: effect, p < 0.05

Stability of baseline measurements: The baseline measurements of percent duration moving changed across the stimulus presentations (F_21,84 _= 1.82p < 0.0296; Figure 5).

Percent duration moving was not affected by the visual stimuli on day 1 (Stimulus test periods: F_6,24 _= 2.06, p < 0.0967; Observation periods: F_2,8 _= 2.23, p < 0.1698; Stimulus test periods by Observation periods: F_12,48 _= 1.43, p < 0.1860).

On day 2, the percent duration moving, averaged over the three observation periods of the acoustic stimulus presentations, tended to change across stimuli (Stimulus test periods: F_6,24 _= 2.43, p < 0.0563). Presentation of these stimuli did not affect the percent duration moving (Observation periods: F_2,8 _= 1.87, p < 0.2151; Stimulus test periods by Observation periods interaction: F_12,48 _= 0.80, p < 0.6518).

On the third day, the average percent duration moving was not different between stimulus presentations (Stimulus test periods: F_7,28 _= 1.50, p < 0.2063). However, presentation of the combinations of visual and acoustic stimuli lead to a drop in the percent duration moving (Observation periods: F_2,8 _= 4.67, p < 0.0452; Stimulus test periods by Observation periods interaction: F_14,56 _= 1.29, p < 0.2424).

### Overflight of a Chinook helicopter on day 6

When the goats were tested on day 6 the heart rate was on average slightly higher than on the previous days, maybe as response to transferring the animals to the pasture (Figure 3). Unexpectedly, the two overflights of the Chinook helicopter had no effects on the heart rate (Stimulus test periods: F_2,6 _= 1.81, p < 0.2431; Observation periods: F_2,6 _= 0.55, p < 0.6007, Stimulus test periods by Observation periods interaction: F_4,12 _= 0.55, p < 0.7013).

### Behavioral reactions during flyover of the Chinook helicopter (day 6)

**Figure 6:**
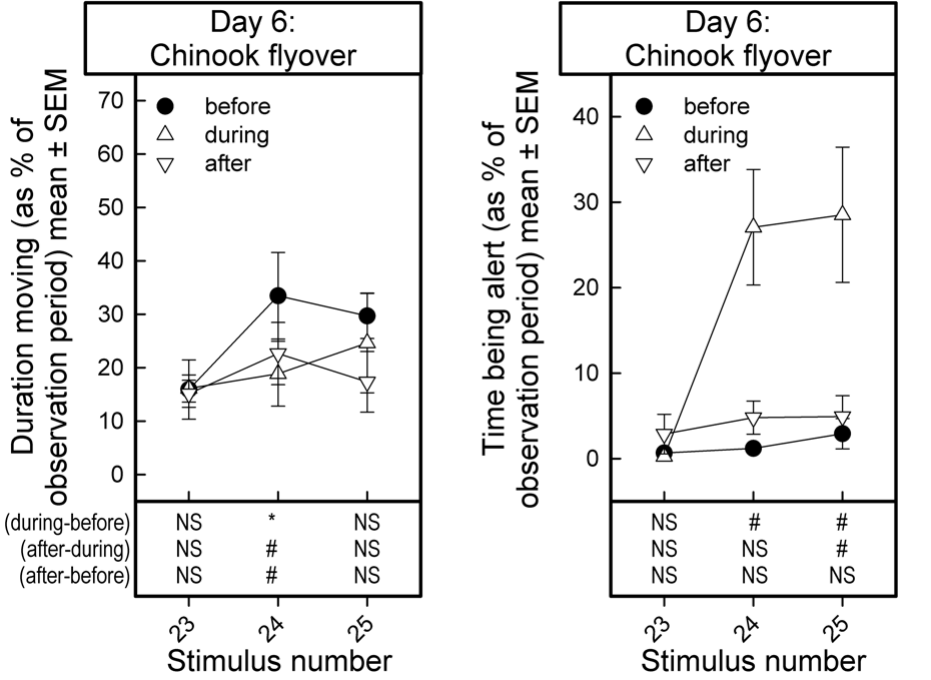
**Average duration moving and time being before, during and after stimulus presentation (flyover of a Chinook helicopter) in dairy goats**. Data are depicted as mean ± SEM. Note that the data of only 3 goats were available for analyzing the effects of the helicopter overflights on alertness of the goats. Results of one-sample t-statistics on the difference scores between values (during minus before), (after minus during), and (after minus before) stimulus presentation are summarized in the lower part of the figures. The list of stimuli presented is shown in Table 1. NS: no effect, p > 0.10; #: marginal effect, 0.10 > p > 0.05; *: significant effect, p < 0.05

*Duration moving *(Figure 6, left panel): The overflight of the Chinook helicopter did not affect the duration moving (as % of the observation periods) (Stimulus test periods: F_2,8 _= 1.68, p < 0.2457; Observation periods: F_2,8 _= 3.21, p < 0.0947; Stimulus test periods by Observation periods interaction: F_4,16 _= 1.30, p < 0.3130). However, one sample t-statistics suggest that the duration moving decreased during the first flyover of the Chinook helicopter at the height of 75 m (difference score of % duration moving during flyover and duration moving before flyover: t_4 _= 3.48, p < 0.025; Figure 6, left panel). Although visual inspection of Figure 6 suggests that a similar effect occurred during the flyover at 50 m, this impression was not confirmed statistically.

*Alertness: *Unfortunately, only the level of alertness of three of the five goats could be observed and scored reliably. The overflight of the Chinook strongly increased the level of alertness of the goats (Stimulus: F_2,4 _= 4.80, p < 0.0865; Observation periods: F_2,4 _= 42.84, p < 0.002; Stimulus by Observation periods interaction: F_4,8 _= 4.71, p < 0.0301). The alertness dropped to baseline levels after overflight of the helicopter, i.e. the effect of overflying was of a short duration (Figure 6, right panel).

The decrease in the time moving was accompanied by a marginal increase in the time being alert (expressed as % of time of the observation periods) during flyover of the Chinook helicopter at 75 m and 50 m (difference score during minus before flyover at 75 m: t_2 _= 4.08, 0.10 > p > 0.05; at 50 m: t_2 _= 3.01, 0.10 > p > 0.05). The level of alertness dropped to the pre-stimulus level in the period following the flyover of the helicopter.

During flyover of the Chinook helicopter, the goats ran in their enclosure, and one goat tried to escape during the flyover of the Chinook at 50 m altitude.

## Discussion

In the present study, we used a number of physiological and behavioral measures to assess the effects of simulated and real life stimuli associated with flyover of helicopters, and with a passing tractor. Presentation of simulated stimuli in a controlled environment is considered as an approach that may facilitate the interpretation of results [22]. The group of dairy goats showed hardly any signs of a stress response or of increased emotional reactivity. Both physiologically and behaviorally they barely responded to presentation of a series of visual or acoustic stimuli and the combination of visual and acoustic stimuli. Unexpectedly, they even did not react with a physiological response (increase of salivary cortisol and heart rate) to the flyover of a Chinook helicopter at 50 m, which produced a peak noise intensity of 110 dB. The overflight produced a noise that was even impressive to the human observers.

### Fear versus anxiety

The increased alertness observed in response to stimulus presentations may be an index of anxiety, whereas the attempt of one goat to escape during the flyover of a Chinook at the altitude of 50 m may be an expression of a fear response [7,10,13]. Increased alertness has also been observed as primary response of feral goats to the sight and sound of helicopters [5].

### Effects of previous experience

All goats were purchased from the same commercial supplier and had been reared under the same conditions. The goat farm lies in an area in which helicopters are allowed to fly at low altitude. It is unknown whether the animals had previous experience with helicopter and/or aircraft noise. It is likely that they had been exposed to auditory, but not visual stimuli produced by airplanes and helicopters before, because the animals were kept in a stable all year round.

Also, we cannot exclude that the testing program of the present study acted as 'exposure therapy', which is a desensitization approach. In this approach, the animal is exposed to the putative anxiety producing event(s) under strictly controlled conditions, and the animal learns that the putative fear-producing stimulus is without effect [23]. As a consequence, the intensity of an anxiety reaction fades away or ceases completely.

We presented stimuli of increasing intensity on each day of testing. Increasing the intensity in a series of stimulus presentations may have facilitated adaptation to these stimuli.

### Sensitivity of the experimental approach to detect physiological and behavioral reactions

It was unexpected that we didn't find any statistically reliable increase in cortisol, heart rate and recorded behavior; an increase from baseline for heart rate and salivary cortisol would be indicative for a physiological stress response (a decrease in both measures in response to the different stimuli is considered as highly unlikely) and an increase in mean velocity moving and percent time moving would be indicative of escape/flight behavior, whereas a decrease (possibly to zero) would be indicative of a freezing response. Therefore we checked the number of animals that are needed to detect a change (reduction or increase) from baseline, depending on the direction and size of changes expected from published data. To this end, we estimated the average baseline values and standard deviations for salivary cortisol, heart rate, average velocity moving, and percent time moving of the observation period. Using these data, we calculated power analyses (t-tests for dependent samples, with α = 0.05, β = 80, using G*Power, version 3.1.2 [24,25]) to determine the number of animals needed to detect a decrease or increase of in these measures in reaction to the presentation of the stimuli (Table 2).

**Table 2:** Results of power analyses for salivary cortisol, heart rate, and duration moving

Salivary cortisol (baseline standard deviation: 0.23)			Heart rate (baseline standard deviation: 12.01)			Duration moving (baseline standard deviation: 22.97)		
**%**	**mean**	**N**	**%**	**mean**	**N**	**%**	**mean**	**N**

350	1.51	**3**	200	158.20	**2**	300	79.02	**4**
300	1.29	**3**	180	142.38	**3**	260	68.48	**5**
250	1.08	**4**	160	126.56	**3**	220	57.95	7
200	0.86	**5**	140	110.74	**3**	180	47.41	12
150	0.65	10	120	94.92	6	140	36.87	40

100	0.43		100	79.10		100	26.34	

80	0.34	54	90	71.19	16	80	21.07	152
60	0.26	15	80	63.28	6	60	15.80	40
40	0.17	8	70	55.37	**4**	40	10.54	19
#			60	47.46	**3**	20	5.27	12

Power analyses were performed for α = 0.05 and β = 0.80.

#: lower cortisol values not considered, because average decreases below detection limit.

%: change in percent from baseline value, with baseline value set to 100%

The means and standard deviations of the baseline measure, i.e. of the 12-minute observation periods before stimuli were used for the power calculations. The daily baseline of cortisol was calculated as mean of the cortisol measurements preceding presentation of the first non-neutral stimulus (Table 1, last column).

*Salivary cortisol: *from published data of transport stress studies, we expected an acute 3- [26] to 8-fold [27] increase in salivary cortisol with respect to the baseline values in response to the visual and auditory stimuli. However, cortisol levels increased only transiently and returned rapidly, within about 1 hour, to baseline values [26-28]. A reduction of salivary cortisol in response to the stress-inducing stimuli was considered as unlikely. Based on our baseline measurements, a group of 5 goats would have been enough to detect an at least 2-fold increase of salivary cortisol.

*Heart rate: *it has been reported that the heart rate in goats exposed to isolation stress increases approximately 60% with respect to baseline [29]. Feeding and physical activity have also been reported to increase the heart rate in goats. Walking alone can increase the heart rate by approximately 60% [30]. Based on the baseline heart rate measurements in the present study, a group of 3 goats would have been enough to detect an increase in heart rate in response to the presentation of stimuli of at least 40%.

*Mean duration moving: *the goats were not very active in their enclosures during baseline measures. They moved during approximately 28% of these observation periods. If stimulus presentation induced freezing, the duration moving should approach 0%, whereas an increase of > 300% would indicate that the goats were moving nearly during the entire observation period after presentation of stimuli. Based on the baseline measurements in the present study, freezing would not reliably have been detected with a group of 5 goats. However, an activating effect to 260% or more of baseline would have been detected with 5 goats. In that case, the animals would be moving during most of the observation period after stimulus presentation.

Head [31] did not observe any signs of startle, retreat of freezing behavior by cows that were exposed to simulated jet noise (maximum dBA: 113.6). Also, the animals did not show any change in the response to the sound over a testing period of 21 days.

Dairy goats and cows are usually kept in environments with various sources of noise, such as traffic, machinery, aircrafts and helicopters. As long as the intensity of these noised do not exceed the normal background noise level, one may not expect any reaction to these stimuli. According to Manci and colleagues [1] sound levels considerably higher than 90 dB are needed to evoke a retreat, freezing or startle response in mammals. However, before overt behavioral reactions are visible, physiological markers may already respond to higher noise levels, i.e. the stimuli may evoke subtle reactions.

### Individual and herd reactions

Individuals differ in their styles of coping with environmental demands and challenges, i.e. in their responses to social and non-social challenging or novel situations. These reactions appear to be relatively stable across life history [32]. Coping style is a concept that is closely related to the concept of temperament. Koolhaas et al. [33] distinguish two coping styles, or stress response patterns: proactive (or active) coping, and reactive coping. Proactive coping is characterized by an intrinsically driven, rigid response (e.g. aggression), whereas reactive coping is characterized by responses that are triggered by the environment. The reactive coping style is believed to be more flexible and adaptive [34]. Following the terminology by Réale and colleagues [32], goats may be characterized along the temperament dimension bold - shy (fearful). An overflying helicopter may increase alertness, but may also trigger a (panic) flight reaction.

Dairy goats are generally kept in herds and the physiological and behavioral reactions measured for individually housed animals in the present study may not be representative for goats in a herd. The variation between individuals for in a herd may be large, and may be evolutionary stable, probably because this mix has adaptive value for the herd. Temperament trait in goats appear to be relatively resistant to change [35]. A few shy animals may largely determine the reaction of the entire herd to stimuli that are perceived as aversive or threatening, because flight reactions are facilitated socially, i.e. they may take the lead in these situations. It has been reported that leadership is less well defined in herds of goats than it is in flocks of sheep [19]. The detection of a threat by a few alert animals and their reaction, e.g. panic flight, will most likely result in a flight reaction of the whole group [5]. Consequently, a measure to address the problem of panic flight, where it exists, may be to remove individuals from the herd, which show a strong behavioral reaction to the noise of overflying helicopters, or any other stimulus that elicits an overt stress or fear reaction. Such a measure is not needed if the animals show a freezing response, because freezing is not expected to increase the incidence of self injury or of injuring other members of the herd, whereas a panic flight response does. A shelter within reach may provide a "safe" place to escape to in case of visual and acoustic stimuli that may be perceived as threatening and that elicit a fear reaction, such as flight.

On the other hand, the mild or absent reaction to strong visual and acoustic stimuli seen in the present study in individually housed goats, which previously had been presented stimuli of increasing intensity, may open perspectives for "treating" herds that may show a stress or fear response to intense acoustic and/or visual stimuli [18]. Through gradual exposure to the increasingly higher levels of the stimuli, the animals may habituate to these stimuli [1].

## Conclusions

In line with studies performed with other ruminants, goats may be quite resistant to the effects of intense, adverse visual and acoustic stimuli such as the sight and noise of overflying helicopters.

## Competing interests

This research was commissioned and sponsored by the Royal Netherlands Air Force (RNLAF). It was performed by the National Aerospace Laboratory (NLR, Amsterdam, The Netherlands) in cooperation with Biomedical Research, Animal Sciences Group of Wageningen University and Research Center (WUR, Lelystad, The Netherlands).

## Authors' contributions

TS, JM, MJ and HD designed the study. JM supervised the experiments. JM and FJS analyzed the data and prepared the manuscript. TS, MJ and HD contributed to, read and approved the final manuscript.

## References

[B1] Manci KM Gladwin DN Villella R Cavendish MG Effects of aircraft noise and sonic booms on domestic animals and wildlife: a literature synthesis US Fish and Wildlife Service, National Ecology Research Center 1988 Fort Collins, CO (USA) 88

[B2] Head HH Kull RC Jr Campos MS Bachman KC Wilcox CJ Cline LL Hayen MJ Milk yield, milk composition, and behavior of holstein cows in response to jet aircraft noise before milking Journal of Dairy Science 1993 76 1558 1567 10.3168/jds.S0022-0302(93)77489-5

[B3] Pepper CB Nascarella MA Kendall RJ A review of the effects of aircraft noise on wildlife and humans, current control mechanisms, and the need for further study Environmental Management 2003 32 418 432 10.1007/s00267-003-3024-4 14986892

[B4] Armas NM Military aviation noise and its effects on domesticated and wild animals Penn State Environmental Law Review 2004 12 367 388

[B5] Tracey JP Fleming PJS Behavioural responses of feral goats (*Capra hircus*) to helicopters Applied Animal Behaviour Science 2007 108 114 128 10.1016/j.applanim.2006.10.009

[B6] Heffner RS Heffner HE Hearing in large mammals: sound-localization acuity in cattle (*Bos taurus*) and goats (*Capra hircus*) Journal of Comparative Psychology 1992 106 107 113 10.1037/0735-7036.106.2.107 1600717

[B7] Catherall DR How fear differs from anxiety Traumatology 2003 9 76 92 10.1177/153476560300900202

[B8] Lang PJ Davis M Ohman A Fear and anxiety: animal models and human cognitive psychophysiology Journal of Affective Disorders 2000 61 137 159 10.1016/S0165-0327(00)00343-8 11163418

[B9] Davis M Are different parts of the extended amygdala involved in fear versus anxiety? Biological Psychiatry 1998 44 12 1239 1247 10.1016/S0006-3223(98)00288-1 9861467

[B10] McNaughton N Corr PJ A two-dimensional neuropsychology of defense: fear/anxiety and defensive distance Neuroscience and Biobehavioral Reviews 2004 28 285 305 10.1016/j.neubiorev.2004.03.005 15225972

[B11] Blanchard RJ Blanchard DC Attack and defense in rodents as ethoexperimental models for the study of emotion Progress in Neuropsychopharmacology and Biological Psychiatry 1989 13 Suppl S3 S14 10.1016/0278-5846(89)90105-X 2694228

[B12] McNaughton N Cognitive dysfunction resulting from hippocampal hyperactivity--a possible cause of anxiety disorder? Pharmacology Biochemistry and Behavior 1997 56 603 611 10.1016/S0091-3057(96)00419-4 9130284

[B13] Ohl F Arndt SS van der Staay FJ Pathological anxiety in animals Veterinary Journal 2008 175 18 26 10.1016/j.tvjl.2006.12.013 17321766

[B14] Boissy A Fear and fearfulness in animals The Quarterly Review of Biology 1995 70 165 191 10.1086/418981 7610234

[B15] Rosen JB Schulkin J From normal fear to pathological anxiety Psychological Review 1998 105 325 350 10.1037/0033-295X.105.2.325 9577241

[B16] Bayne P Harden B Pines K Taylor U Controlling feral goats by shooting from a helicopter with and without the assistance of ground-based spotters Wildlife Research 2000 27 517 523 10.1071/WR99059

[B17] Côté SD Mountain goat responses to helicopter disturbance Wildlife Society Bulletin 1996 24 4 681 685

[B18] Kempf N Hüppop O Auswirkungen von Fluglärm auf Wildtiere: ein kommentierter Überblick Journal für Ornithologie 1996 137 101 113

[B19] Miranda-de la Lima GC Mattiello S The importance of social behaviour for goat welfare and livestock farming Small Ruminant Research 2010 90 1 10 10.1016/j.smallrumres.2010.01.006

[B20] Noldus LPJJ Spink AJ Tegelenbosch RAJ EthoVision: a versatile video tracking system for automation of behavioral experiments Behavior Research Methods, Instruments, & Computers 2001 33 398 414 10.3758/bf03195394 11591072

[B21] Spink AJ Tegelenbosch RAJ Buma MOS Noldus LPJJ The EthoVision video tracking system - a tool for behavioral phenotyping of transgenic mice Physiology & Behavior 2001 73 731 744 10.1016/s0031-9384(01)00530-3 11566207

[B22] Manteca X Deag JM Use of physiological measures to assess individual differences in reactivity Applied Animal Behaviour Science 1993 37 265 270 10.1016/0168-1591(93)90116-7

[B23] Todd JT Pietrowski JL Richard DCS, Lauterbach D Animal models of exposure therapy: a selective review Handbook of exposure therapy 2007 Academic Press 29 59 full_text

[B24] Erdfelder E Faul F Buchner A GPOWER: a general power analysis program Behavior Research Methods, Instruments, & Computers 1996 28 1 11

[B25] Faul F Erdfelder E Lang AG Buchner A G*Power 3: a flexible statistical power analysis program for the social, behavioral, and biomedical sciences Behavior Research Methods, Instruments, & Computers 2007 39 175 191 10.3758/bf03193146 17695343

[B26] Kannan G Terrill TH Kouakou B Gazal OS Gelaye S Amoah EA Samaké S Transportation of goats: effects on physiological stress responses and live weight loss Journal of Animal Science 2000 78 1450 1457 10875626 10.2527/2000.7861450x

[B27] Greenwood PL Shutt DA Salivary and plasma cortisol as an index of stress in goats Australian Veterinary Journal 1992 69 7 161 163 10.1111/j.1751-0813.1992.tb07501.x 1445079

[B28] Nwe TM Hori E Manda M Watanabe S Significance of catecholamines and cortisol levels in blood during transportation stress in goats Small Ruminant Research 1996 20 129 135 10.1016/0921-4488(95)00781-4

[B29] Aschwanden J Gygax L Wechsler B Keil NM Cardiac activity in dairy goats whilst feeding side-by-side at two different distances and during social separation Physiology and Behavior 2008 95 641 648 10.1016/j.physbeh.2008.09.016 18851984

[B30] Berhan T Puchala R Goetsch AL Merkel RC Effects of walking speed and forage consumption on energy expenditure and heart rate by Alpine does Small Ruminant Research 2006 63 119 124 10.1016/j.smallrumres.2005.02.012

[B31] Head HH Behavior and milk yield responses of dairy cattle to simulated jet aircraft noise 1992 Armstrong Laboratory, Wright-Patterson Air Force Base, Ohio

[B32] Réale D Reader SM Sol D McDougall PT Dingemanse NJ Integrating animal temperament within ecology and evolution Biological Research 2007 82 291 318 10.1111/j.1469-185X.2007.00010.x 17437562

[B33] Koolhaas JM Korte SM de Boer SF van der Vegt BJ van Reenen CG Hopster H de Jong IC Ruis MAW Blokhuis HJ Coping styles in animals: current status in behavior and stress physiology Neuroscience and Biobehavioral Reviews 1999 23 925 935 10.1016/S0149-7634(99)00026-3 10580307

[B34] Campbell T Lin S deVries C Lambert K Coping strategies in male and female rats exposed to multiple stressors Physiology and Behavior 2003 78 495 504 10.1016/S0031-9384(03)00033-7 12676287

[B35] Lyons DM Price EO Moberg GP Individual differences in temperament of domestic dairy goats: constancy and change Animal Behaviour 1988 36 1323 1333 10.1016/S0003-3472(88)80201-X

